# Supplementary material for: Advanced deep learning and transfer learning approaches for breast cancer classification using advanced multi-line classifiers and datasets with model optimization and interpretability
Source: PeerJ Comput Sci. 2025 Jul 9;11:e2951. doi: 10.7717/peerj-cs.2951 (PMC12453660; doi:10.7717/peerj-cs.2951)
Supplement: Supplemental Information 1 [file peerj-cs-11-2951-s001.docx]

**README: Breast Cancer Diagnosis Using Machine Learning**

**Project Overview**

This project focuses on developing machine learning models to classify breast cancer as benign or malignant using the **Breast Cancer Wisconsin Dataset**. The models explored include **Random Forest**, **XGBoost**, and **Deep Neural Networks (DNN)**. This repository includes the code, data preprocessing steps, and results analysis necessary for reproducibility.

**Prerequisites**

**Hardware Requirements**

- Operating System: Ubuntu 20.04 LTS or equivalent
- CPU: Intel Core i9 or higher
- GPU: NVIDIA Tesla V100 or equivalent (for DNN training)
- RAM: 64 GB or more
- Storage: At least 2 TB SSD

**Software Requirements**

- Python 3.8 or higher
- Libraries:
  - Data Processing: NumPy (>= 1.21), Pandas (>= 1.3)
  - Visualization: Matplotlib (>= 3.4), Seaborn (optional)
  - Machine Learning: scikit-learn (>= 0.24), XGBoost (>= 1.4.2)
  - Deep Learning: TensorFlow (>= 2.6), Keras (>= 2.6), or PyTorch (optional)
  - Optimization: Optuna (optional for hyperparameter tuning)
  - Explainability: SHAP, LIME

Install the required libraries using:

bash

Copy code

pip install -r requirements.txt

**Dataset**

**Source**

The dataset used in this study is the **Breast Cancer Wisconsin Dataset**, available at:

- [Kaggle](https://www.kaggle.com/datasets/uciml/breast-cancer-wisconsin-data)
- [UCI Machine Learning Repository](https://archive.ics.uci.edu/ml/datasets/Breast+Cancer+Wisconsin+(Diagnostic))

**Description**

- **Instances**: 569
- **Features**: 30 numerical features describing cell nuclei characteristics
- **Target Variable**: Diagnosis (M for malignant, B for benign)

**Folder Structure**

plaintext

Copy code

|-- data/

| |-- breast_cancer.csv # Original dataset

|-- src/

| |-- preprocessing.py # Data preprocessing scripts

| |-- train_models.py # Model training scripts

| |-- evaluation.py # Performance evaluation

|-- notebooks/

| |-- exploratory_analysis.ipynb # Jupyter notebook for EDA

|-- results/

| |-- performance_metrics.csv # Evaluation results

| |-- visualizations/ # Plots (ROC curves, feature importance)

|-- README.md # Project overview

|-- requirements.txt # List of required Python packages

|-- LICENSE # Licensing information (MIT/GPL/etc.)

**Setup and Implementation**

1. **Clone the Repository**
   Clone the repository to your local system:

bash

Copy code

git clone https://github.com/your-repo-name/breast-cancer-ml.git

cd breast-cancer-ml

1. **Prepare the Dataset**
   Download the dataset from the provided sources and place it in the data/ directory.
2. **Install Dependencies**
   Use the following command to install all required libraries:

bash

Copy code

pip install -r requirements.txt

1. **Run Data Preprocessing**
   Preprocess the dataset by executing:

bash

Copy code

python src/preprocessing.py

1. **Train Machine Learning Models**
   Train the models by running:

bash

Copy code

python src/train_models.py

1. **Evaluate Models**
   Assess model performance with:

bash

Copy code

python src/evaluation.py

1. **Analyze Results**
   Open the Jupyter notebook for exploratory and post-evaluation analysis:

bash

Copy code

jupyter notebook notebooks/exploratory_analysis.ipynb

**Key Features**

1. **Automated Preprocessing**: Handles missing values, encodes labels, and normalizes features.
2. **Comprehensive Model Training**: Implements Random Forest, XGBoost, and Deep Neural Networks with hyperparameter tuning.
3. **Performance Metrics**: Calculates accuracy, precision, recall, F1-score, and ROC-AUC with visualizations.
4. **Explainability**: Provides SHAP and LIME explanations for model predictions.

**Notes on Reproducibility**

1. **Random Seeds**: Set random seeds for all libraries to ensure deterministic results.

python

Copy code

import numpy as np

import tensorflow as tf

import random

random.seed(42)

np.random.seed(42)

tf.random.set_seed(42)

1. **Hardware Constraints**: For large DNNs, ensure adequate GPU resources are available.
2. **Supplementary Files**: Include the complete code (Supplementary File 1) and additional documentation for preprocessing (Supplementary File 2).
