# Supplementary material for: Advanced deep learning and transfer learning approaches for breast cancer classification using advanced multi-line classifiers and datasets with model optimization and interpretability
Source: PeerJ Comput Sci. 2025 Jul 9;11:e2951. doi: 10.7717/peerj-cs.2951 (PMC12453660; doi:10.7717/peerj-cs.2951)
Supplement: Supplemental Information 4 [file peerj-cs-11-2951-s004.docx]

**Supplementary File 3:** Performance Metrics for Breast Cancer Classification Models (Including Confidence Intervals, ROC-AUC, and p-values)

| **Model** | **Accuracy (95% CI)** | **Precision (Benign/**  **Class 0)**  **(95% CI)** | **Precision (Malignant/**  **Class 1)**  **(95% CI)** | **Recall (Benign/**  **Class 0)**  **(95% CI)** | **Recall (Malignant/**  **Class 1)**  **(95% CI)** | **F1-score (Benign/**  **Class 0)**  **(95% CI)** | **F1-score (Malignant/**  **Class 1)**  **(95% CI)** | **ROC-AUC** | **p-value** |
| --- | --- | --- | --- | --- | --- | --- | --- | --- | --- |
| **Random Forest** | 96.5% (93.1–98.6%) | 0.96 (0.92–0.98) | 0.98 (0.94–0.99) | 0.99 (0.96–1.00) | 0.93 (0.87–0.97) | 0.97 (0.94–0.99) | 0.95 (0.90–0.98) | 0.98 | 0.010 |
| **XGBoost** | 97.4% (94.2–99.1%) | 0.97 (0.93–0.99) | 0.98 (0.95–0.99) | 0.99 (0.96–1.00) | 0.95 (0.90–0.98) | 0.98 (0.95–0.99) | 0.96 (0.93–0.98) | 0.99 | 0.005 |
| **Deep Neural Net** | 98.0% (95.1–99.5%) | 0.97 (0.94–0.99) | 1.00 (0.97–1.00) | 1.00 (0.97–1.00) | 0.95 (0.90–0.98) | 0.99 (0.97–1.00) | 0.98 (0.95–0.99) | 0.995 | <0.001 |
